# Cross-species comparisons in a unified medium suggest broadly stable glycosome-linked enzyme levels under nutrient and oxygen variation

**DOI:** 10.3389/fpara.2026.1823935

**Published:** 2026-06-15

**Authors:** Hina Durrani, James A. Bjork, Aaron B. Clarke, Jackson T. Steinbach, Sara L. Zimmer

**Affiliations:** 1 Swenson College of Science and Engineering, University of Minnesota Duluth, Duluth, MN, United States; 2 Department of Biomedical Sciences, University of Minnesota Medical School, Duluth, MN, United States

**Keywords:** evolution, glycosome, *Leishmania*, metabolic remodeling, peroxisome, trypanosome

## Abstract

**Introduction:**

Kinetoplastids are one of two groups of euglenozoans that have compartmentalized the enzymes of metabolic pathways, including glycolysis, into a peroxisome-derived organelle called a glycosome. Several ideas have emerged regarding the evolutionary pressures that drove and are maintaining compartmentalization of these typically cytosolic enzymes. For example, their compartmentalization might result in superior efficiency in remodeling enzyme relative abundances in response to changing environmental conditions. We wished to empirically test the merits of this explanation. We began with the presumption that the abundances of glycosome-compartmentalized enzymes are broadly affected by changes in extracellular environment, similar to how they vary between life stages of the model kinetoplastid *Trypanosoma brucei*. Our hypothesis was that abundances of glycosome-localized enzymes in multiple kinetoplastids would alter as a result of their extracellular environment.

**Methods:**

Six different kinetoplastid species, including both dixenous and monoxenous parasites, were evaluated in culture for their response to extracellular nutrient and oxygen availability in terms of their expression of glycosome-localized enzymes. We analyzed their growth rates and utilized immunoblotting to measure abundances of glycosome-localized or partially-localized enzymes and cytosolic enolase in the different culture conditions.

**Results:**

We found the tested enzyme abundances to be largely consistent. Oxygen availability and slow removal of nutrients from the extracellular environment resulted in only minor changes in enzyme abundance. Only abrupt replenishment of culture nutrients resulted in enzyme abundance responses – typically a modest decrease in both cytosolic enolase and the glycosome-localized enzymes.

**Discussion:**

Results suggest that there is little evidence that parasites respond to external nutrient or oxygen availability through alterations in relevant enzyme abundances, at least absent life cycle transitions. Thus, our study does not provide support for the idea that efficient and coordinated remodeling of ratios of metabolic enzymes was an evolutionary driver of metabolic enzymes’ compartmentalization in the glycosome. Further, it suggests that it may be relevant to test whether posttranslational modifications and/or changes to enzyme localization are mechanisms trypanosome utilize to adjust specific metabolic activity when needed.

## Introduction

Theoretical benefits of the evolution, maintenance, and innovation of cellular compartmentalization include optimized enzyme activity, the containment or neutralization of metabolic by-products, minimal intermediate substrate diffusion, and protection from inhibitors (Bar-Peled and Kory, 2022). However, verifying these advantages as drivers of cellular compartmentalization is challenging due to the immense time scales involved, the complexity of eukaryotic cell structure, and limited organisms in which they have been studied across the eukaryotic tree of life. Kinetoplastids are a group of unicellular flagellates within the Euglenozoa, a radically diverged branch of eukaryotes from those that are frequently analyzed (Kostygov et al., 2021). Most kinetoplastids identified thus far are parasites of insects, although dixenous life cycles have independently arisen within the group several times (Lukeš et al., 2014). Kinetoplastids, along with the diplonemids, comprise the Glycomonada, possessing a unique version of a peroxisome with highly altered contents. These organelles, called glycosomes, house enzymes of major metabolic pathways that are cytosolic in well-studied eukaryotic systems (Allmann and Bringaud, 2017; Andrade-Alviárez et al., 2022). Thus, they offer an excellent model for examining the drivers, benefits and outcomes of partitioning metabolic pathways within specific cellular locales.

The glycosome was named for the initial seven enzymes of the glycolysis pathway that are localized in its matrix. Several theories have been proposed to explain the localization of glycolysis and parts of other metabolic processes that are uniquely compartmentalized there. One theory suggests that this spatial organization circumvents feedback inhibition that typically regulates glycolysis. Notably, while hexokinase (HK) and phosphofructokinase (PFK) are typically downregulated by their end products, the African trypanosome *Trypanosoma brucei* orthologues (TbHK and TbPFK) do not show this inhibitory response (Nwagwu and Opperdoes, 1982). The ATP used by initial enzymes of glycosolysis is replenished by ATP generated further along the same pathways. Without compartmentalization, unregulated TbHK localized with downstream enzymes in the cytosol could lead to uncontrolled glucose phosphorylation and ATP exhaustion, culminating in a lethal “turbo-explosion” (Haanstra et al., 2008). However, broader studies now suggest that extreme loss of regulatability of PFK is not universal among kinetoplastids (Fernandes et al., 2020). Thus, loss of regulatability of glycolysis enzymes may be a consequence rather than a driver of their compartmentalization (Gualdrón-López et al., 2012). Another idea is that compartmentalization streamlines glycolysis by ensuring proximity of enzymes within the same pathway to facilitate hand-off of metabolic intermediates (Opperdoes, 1987). However, this was deemed unlikely through a computational kinetic approach (Bakker et al., 2000) and growing knowledge of the full range of metabolic flux among kinetoplastids (Gualdrón-López et al., 2012). The most persistent theory is that the compartmentalization of these specific enzymes to glycosomes evolved as a competitive advantage by allowing rapid, coordinated remodeling of their abundances. Mechanistically this would involve autophagic degradation of glycosomes, a phenomenon analogous to peroxisomal “pexophagy” (Herman et al., 2008), followed by the genesis of glycosomes with altered enzymatic profiles (Andrade-Alviárez et al., 2022). This seems a particularly efficient way to coordinate changes to gene expression in response to environmental cues.

This final theory is largely based on the enzyme composition of the *T. brucei* glycosomal matrix that differs radically between its two replicative life stages. Differing *T. brucei* glycosomal matrix compositions are consistent with its metabolic needs in its different host environments (Gualdrón-López et al., 2012). In the gut of its tsetse fly (Glossina spp.) insect vector where glucose is transient, amino acids, especially proline, are key nutrients. In contrast, the parasite in mammals replicates in the glucose-rich bloodstream, relying near-exclusively on glycolysis for energy and ceasing cytochrome-mediated aerobic respiration. Consequently, some proteins linked to the pentose phosphate (PPP) and purine salvage pathways trend toward higher abundance in the *T. brucei* insect vector (procyclic) life stage than in the replicating bloodstream life stage. Conversely, many glycolysis enzymes are strongly upregulated in bloodstream life stage parasites. Complicating the picture, however, is that the individual glycosomes of a cell are not uniform in composition (Bauer et al., 2013; Anderson et al., 2026; Michels and Gualdrón‐López, 2022).

Importantly, *T. brucei* exhibits the most extreme example of life-stage specific metabolic remodeling among studied dixenous kinetoplastids (Tielens and van Hellemond, 2009). It is less clear the extent to which radical metabolic enzyme remodeling is part of adaptation to life stage differences in dixenous kinetoplastids other than *T. brucei*. It is also not clear whether environmental conditions or signals divorced from the context of life stage transitions are sufficient to stimulate remodeling of glycosome-localized enzyme abundances in either monoxenous or dixenous species. Particularly considering that monoxenous species predominate in number, this would have to be true for metabolic remodeling efficiency to be the universal explanation for why a unique group of metabolic enzymes is compartmentalized to the glycosome. If kinetoplastids do not respond to environmental signals in this manner, then this phenomenon in *T. brucei* may instead reflect the extreme situation of a possible general dixenous trypanosomatid adaptation of the glycosome. Our goal is to address whether glycosome compartmentalization of metabolic pathways broadly confer a competitive advantage by enabling efficient metabolic remodeling in response to a changing environment. Change in abundances of glycosome-localized enzymes in response to extracellular change in both monoxenous and dixenous species is a precondition of this possibility. Nutritional environment and oxygen availability are promising environmental factors to consider.

In this study we determined how nutrient and oxygen availability impact the expression of glycosome relevant metabolic pathways in six cultured kinetoplastid species, either monoxenous or tested exclusively in the insect life stage of dixenous species. Unlike the situation between *T. brucei* replicative life stages, none of the environmental perturbations we applied resulted in large scale changes to glycosome-localized enzyme levels. This result was maintained even when comparing enzyme levels under different culture conditions in procyclic *T. brucei*. This finding would suggest that compartmentalization of certain enzymes into glycosomes is not a general mechanism required by these organisms to efficiently remodel protein abundance in response to the environmental changes of nutrient or oxygen availability. Rather, other evolutionary drivers likely play a role in the establishment and continued presence of glycosomes in kinetoplastids and in their sister clade diplonema.

## Materials and methods

### Parasite culture

*T. brucei* 29-13 (Lister 427 29-13 (TetR T7RNAP)), *Trypanosoma cruzi* Sylvio X10 (ATCC 50823), *Leishmania tarentolae* (ATCC 30143), *Leptomonas pyrrhocoris* H10 cells (Votýpka et al., 2012), *Leptomonas seymouri* (ATCC 30220), and *Vickermania ingenoplastis* (ATCC 30259), were maintained in Liver Infused Tryptose (LIT) medium (Castellani et al., 1967) supplemented with 10% FBS, in which either the standard amount (2 g/L), 0.4 g/L, or 0 g/L glucose was added, referred to as standard, low, and no glucose media respectively. To measure growth, cultures were started at 1 X 10^6^ or 2 X 10^6^ cells/ml (depending on experiment) and counted using a hemocytometer. Cultures were maintained between 10^5^ and 10^7^ cells/ml at 27 °C. *T. cruzi*, *L. tarentolae*, *L. pyrrhocoris*, and *L. seymouri* were consistently cultured in media with each designated glucose concentration (three stock cultures per species). *T. brucei* and *V. ingenoplastis* were propagated in standard glucose-containing LIT media and then transitioned to media with low or absent glucose content ten days before experimentation. This preparatory step is necessary as the proliferation rates for these species diminish substantially within approximately three weeks when cultured in glucose-depleted conditions. For experiments under specific conditions of environmental oxygen, cell cultures were subjected to either a low-oxygen environment using a hypoxia chamber (Whitley i2 Instrument Workstation) set at 3% oxygen, or in an incubator with standard atmospheric oxygen levels of 20%. A threshold of 3% oxygen was established for the hypoxia experiments because at levels lower than this, the parasites exhibited either no growth or grew so slowly that collecting sufficient protein samples was not feasible. Temperature and carbon dioxide levels were kept constant between the two oxygen conditions. Cultures were maintained under these specific conditions for six days, with protein samples collected within the controlled environments.

### Medium glucose measurements

Amplex Red Glucose/Glucose oxidase kit (Invitrogen, catalog number: A22189) was used to measure media glucose concentrations according to kit instructions. If medium glucose concentration was measured in a growing culture, parasites were first removed by centrifugation (1200 x g, 5 min).

### Immunoblotting & densitometry

5 x 10^6^ cells were collected by centrifugation (1200 x g, 5 min) washed once with PBS and resuspended in SDS-PAGE loading buffer (250 mM Tris-Cl pH 6.8, 8% SDS, 0.1% Bromophenol blue, 40% Glycerol, 100 mM Dithiothreitol). Cell lysates were separated on a 10% SDS-PAGE gel and transferred to a nitrocellulose membrane. Membranes were Ponceau stained, with the staining pattern scanned into a file for later normalization. Membranes were blocked in 2% non-fat milk in 1 x TBST (10 mM Tris-Base, 150 mM NaCl, 0.05% Tween-20, pH 8.0). After blocking, primary antisera from the Frederic Bringaud group was used in 1:10,000 dilution overnight at 4 °C: *T. brucei* enolase (Rabbit SB763, 1:10,000), *L. mexicana* triosephosphate isomerase (TIM) (Rabbit DO424, 1:10,000), *T. brucei* glucose-6-phosphate dehydrogenase (G6PD) (Rabbit SB952, 1:10,000), *L. mexicana* glyceraldehyde-3-phosphate dehydrogenase (GAPDH) (Rabbit DO422, 1:10,000), and *T. brucei* fructose-1,6-bisphosphatase (FBPase) (Rabbit SB764, 1:10,000). An aldolase antibody generated against the *T. brucei* homologue by the Meredith Morris group was used at the same dilution. Proteins of interest were detected with goat anti-rabbit IgG 800CW as the secondary antibody at 1:10,000 dilution. A Li-Cor Odyssey Fc Imager was used to image and analyze gels and membranes. Densitometric analysis was conducted on a minimum of three independent western blots. Signal was quantified by measuring the integrated density of the protein bands, which were then normalized to the loading control (the densitometry of signal from Ponceau S staining of the relevant lane of the membrane) with ImageJ software. Subsequently, the loading-normalized values were further normalized to the mean density of all bands (grand mean) in replicate blots for each species and protein. The standard error of the mean (SEM) was used to express the variability, which represents the precision of the mean integrated density values derived from the three replicate blots.

## Results

### Multiple kinetoplastid species can replicate in liver infusion tryptose medium

A major advantage of compartmentalizing metabolic pathways to the kinetoplastid glycosome may be to enable rapid remodeling of the affected pathways upon changes to the extracellular environment. If this is the case, we would expect the levels of glycosome compartmentalized enzymes to be responsive to extracellular perturbations. While large life-stage differences in glycosome content have been characterized in at least *T. brucei* (Parsons and Nielsen, 1990; Colasante et al., 2006), with some support for a similar phenomenon in *Leishmania* and *T. cruzi* (Cull et al., 2014; Rosenzweig et al., 2008b; Shiratsubaki et al., 2020), the responsiveness of glycosome contents to changes in kinetoplastids’ extracellular environments is a major gap in knowledge. With this study, we are attempting to empirically test this idea. Understanding that such responses may differ depending on the species investigated, we pursued this question employing both dixenous and monoxenous kinetoplastid species available from a reliable, universally accessible source (ATCC). These were *T. brucei* 29-13, *T. cruzi* Sylvio X/10, *L. tarentolae*, *L. pyrrhocoris*, *L. seymouri*, and *V. ingenoplastis*. This array ensured that kinetoplastids both distantly related and closely related to each other would be represented. Ideally, we would have included a free-living species and glycosome-containing Diplonema, considering that our question concerns both the original and continued driving forces leading to compartmentalization of normally cytosolic enzymes. This is particularly relevant as the process may have initiated in marine species (Andrade-Alviárez et al., 2022). However, we envisioned difficulties related to unified growth conditions and antisera cross-reactivity (see below) for these species. Their non-inclusion here will have to remain a study limitation.

**Figure 1:**
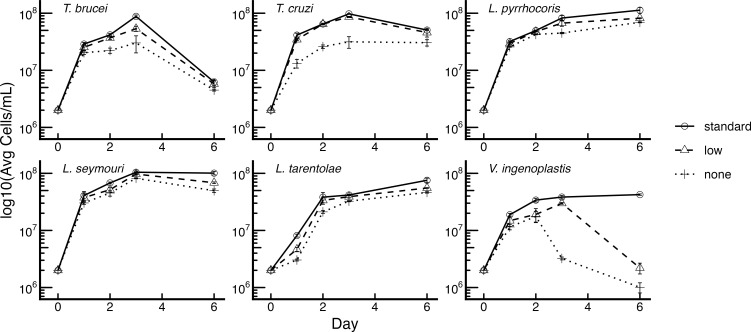
Parasite culture growth rates. The continuous growth of parasites starting from 2 x 10^6^ cells/ml was quantified in LIT media with varying initial glucose concentrations: glucose-rich standard (2 g/L, 11.1 mM), low (0.4 g/L, 2.2 mM), and no glucose (0 g/L, 0.03 mM). Error bars denote standard deviation.

**Figure 2:**
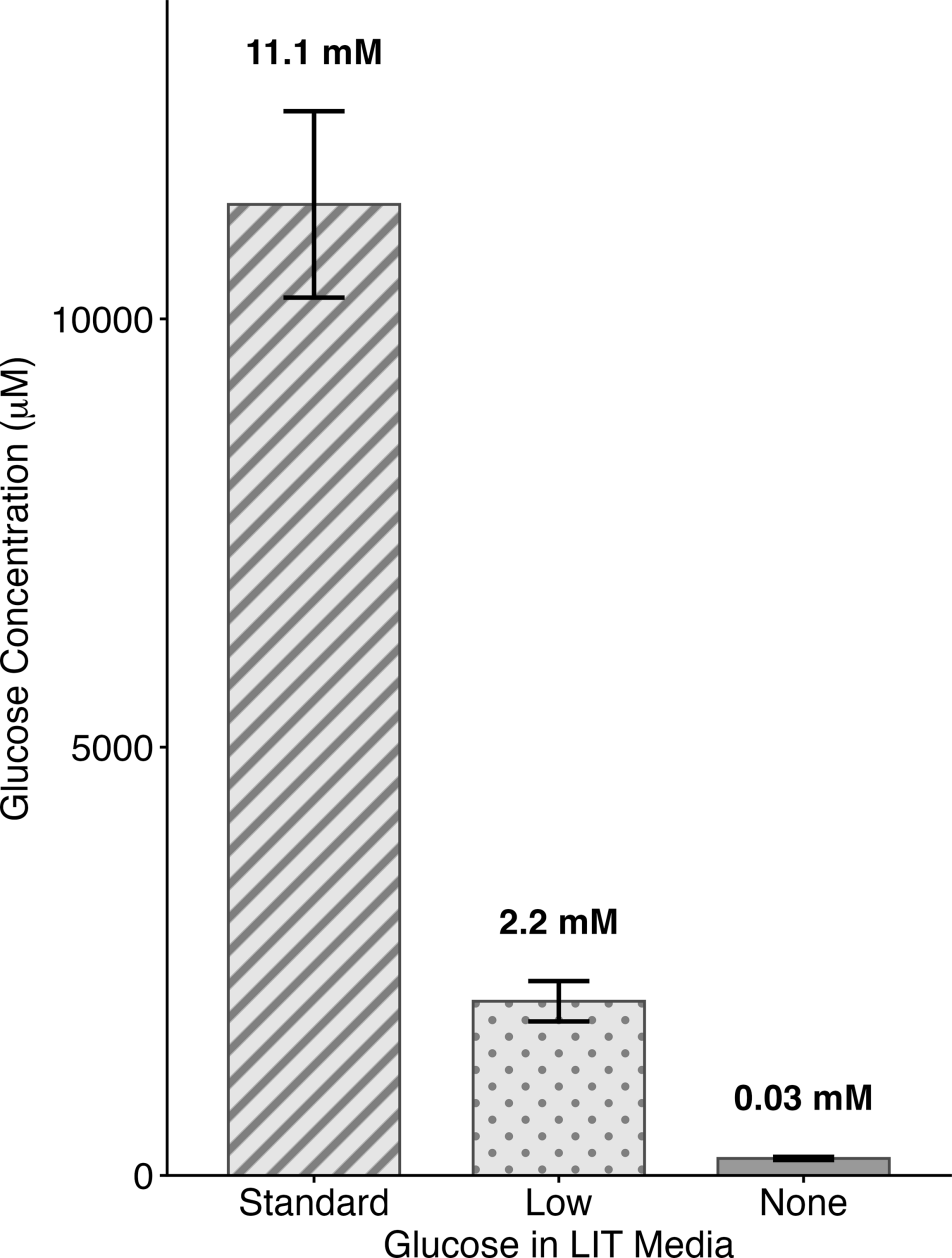
Measured glucose concentration in the three media formulations with added glucose of 2 g/L, 0.4 g/L and 0 glucose (Glucose-rich Standard, Low, and None, respectively), as determined by Amplex Red assay with fluorescent output.

These species have been grown in a variety of culture growth conditions in past studies. However, we found them to all successfully replicate indefinitely in the LIT medium typically used for *T. cruzi* growth with its standard glucose concentration of 2 g/L (Castellani et al., 1967), **Figure 1**. The exception was *T. brucei*, which was maintained in SDM-79 (Brun and Schönenberger, 1979) long term, but could be conditioned to grow in LIT for time periods sufficient for these studies. We then established additional LIT-based culture conditions in which the favored nutritional source of glucose (Bringaud et al., 2006), generally considered in LIT to be higher than biologically relevant, was reduced (arbitrarily to a concentration five-fold less) or removed. To ensure that undefined medium components did not substantially impact medium glucose levels, we measured the actual glucose concentrations of these three media (**Figure 2**). We determined when these species had depleted glucose from the medium during growth to determine appropriate culture time points at which to sample parasite glycosome content. Area under the curve was used to compare species’ utilization of medium glucose, with concentration in *L. tarentolae* and *Leptomonas* species dropping the most dramatically, particularly in LIT with the standard glucose concentration. While all parasites are still exposed to glucose after one day of growth, by the third day of cultivation glucose is no longer present, regardless of starting concentration (**Supplementary Figure 1**). Consequently, sampling time points were established on culture days one, two, and three to capture the active phase of glucose depletion, and on culture day six to assess the protein profile post-depletion when parasites have presumably been relying on amino acids in the medium for storage. Parasite survival at culture day six is expected to be variable depending on the species.

### Glucose stimulates the replication rate of all species

Before analyzing the effect of the extracellular nutrient environment on parasite glycosome contents, we first wanted to assess its impact on the growth of each species. We postulated that species most impacted by lower total provided glucose may be most responsive as to how they distribute flux through various metabolic pathways. It was already known that *T. brucei*, particularly procyclic form 29–13 strain, exhibits accelerated growth in glucose-rich medium (Furuya et al., 2002; Allmann et al., 2014; Wargnies et al., 2018). Our findings corroborate these observations, with a trend toward a stimulatory effect of glucose for all tested species (**Figure 1**). Species varied in their relative growth rates in the three media across the six-day period. *Leptomonas* spp. and *L. tarentolae* showed minor growth rate differences across varying glucose concentrations while *T. brucei*, *T. cruzi*, and *V. ingenoplastis* demonstrated larger differences in growth in glucose-deprived media at some points during the six-day time period. The poor growth of *V. ingenoplastis* in medium without glucose was expected, as mitochondrial respiration-dependent energetic pathways of ATP generation are limited due to its lack of subunits of respiratory complexes III and IV (Opperdoes et al., 2021; Gerasimov et al., 2025). Consistently, the highest concentration of parasites was achieved when the most initial glucose was provided. Even without glucose, all species achieved sufficient growth and/or survival across the six-day time course to allow for experiments requiring protein collection on culture days one, two, three and six. Considering this growth study, *T. cruzi, L. pyrrhocoris, L. seymouri*, and *L. tarentolae* long-term cultures fed every other day were established in each of the three LIT media from which to initiate further experiments. In contrast, *T. brucei* and *V. ingenoplastis* were transitioned to glucose-deprived states (low or no glucose) for ten days before the start of experiments, as their significantly reduced growth rates under these conditions eventually progressed to growth cessation in longer time frames (not shown).

### Depletion of glucose and overall nutrients from culture medium has only minor impacts on enzyme abundances

Growth patterns in **Figure 1** suggest that glucose and general nutrient availability are extracellular signals capable of influencing the abundance of glycosome-compartmentalized metabolic enzymes. Thus, we tested the influence of glucose and starvation on the expression of glycosomal proteins. We grew parasites over the same six-day time course as used to monitor growth, to observe adaptive responses in protein abundances. We then ascertained whether metabolic remodeling of glycosome-localized enzymes is instigated by a cycle of nutrient deprivation within this framework.

As mRNA and protein levels in trypanosomatids are not necessarily well correlated (Vasquez et al., 2014; Smircich et al., 2015; Cortazzo da Silva et al., 2022), we performed densitometric quantification of signals from immunoblot assays to quantify protein expression directly. Thus, our study was limited by the availability of kinetoplastid protein-specific antibodies that exhibited broad cross-reactivity across the parasites used in this study. Antibodies to proteins normally localized to glycosomes have mostly been raised to *T. brucei* orthologs with the exceptions of an antibody raised against *L. mexicana* glyceraldehyde 3-phosphate dehydrogenase, GAPDH. Upon verification of antibody cross-reactivity (**Supplementary Figure 2**) with all or most of our studied species, we were able to detect by immunoblotting three glycosomal bidirectional glycolytic/glyconeogenesis enzymes (Aldolase; GAPDH; and triose-phosphate isomerase, TIM), one glycolytic enzyme localized to the cytosol (Enolase), and one glycosome-localized enzyme from the gluconeogenesis (Fructose 1,6-bisphosphatase, FBPase) pathway. We were regrettably unable to obtain reliable signal with antisera raised against *T. brucei* PFK except in *T. brucei*, and were not able to procure the HK antisera used in prior studies. We did detect in most species the PPP enzyme glucose-6-phosphate dehydrogenase, G6PD) with dual-localization in the cytosol and glycosome. We are not expecting this enzyme to change radically in abundance or activity in the studied species, as evidence suggests its global abundance and activity is only mildly different in the *T. brucei* bloodstream stage cells relative to procyclic stage (e.g (Butter et al., 2013; Cronín et al., 1989). Interestingly, in the case of *V. ingenoplastis*, the G6PD antibody recognized a protein with migration corresponding to a larger size than expected. Its G6PD homologue is present in tandem copies in the genome. Since this genome is not yet annotated, one or both of these copies may possess an N-terminal extension as does *Leishmania infantum* G6PD, although initial inspection does not support this. While FBPase is not detected in the *V. ingenoplastis* genome, the FBPase antibody recognizes a single protein in this species. Other enzymes of gluconeogenesis are present in this genome (Opperdoes et al., 2021), so we assume that FBPase is present in *V. ingenoplastis*. Still, caution should be used in interpreting results for these two enzymes. Cytosolic enolase is detectable in the examined species and served as a control for metabolic remodeling that is unassociated with compartmentalization. We anticipated that if glycosome matrix composition was responsive to extracellular variations in nutrient abundance or composition, the abundances of the glycosome-localized enzymes would differ across experimental collection time points in total protein samples.

Contrary to this expectation, we found that across the tested species, enzyme expression levels appeared largely constant across the six-day gradual nutrient depletion, and this was consistent for all starting glucose conditions. To rigorously evaluate this, for each starting glucose condition we compared normalized immunoblot signals from culture days 2, 3, and 6 with signals on culture day 1, which we consider to be nutrient-replete (**Supplementary Figure 3**). We identified any statistically significant differences through one-way analysis of V= variance (ANOVA) with subsequent application of Dunnett’s multiple comparison test with a p-value threshold of less than 0.05 (**Supplementary Table 1**). Enzyme abundances after six days of culture growth in *T. brucei* or *V. ingenoplastis* should be treated cautiously, as growth curves indicate that substantial cell death may be occurring by this time point. Any observed changes in enzyme abundances may reflect a response to decreasing extracellular levels of any or all decreasing nutrients including glucose, except in the medium condition in which glucose is completely lacking.

**Figure 3:**
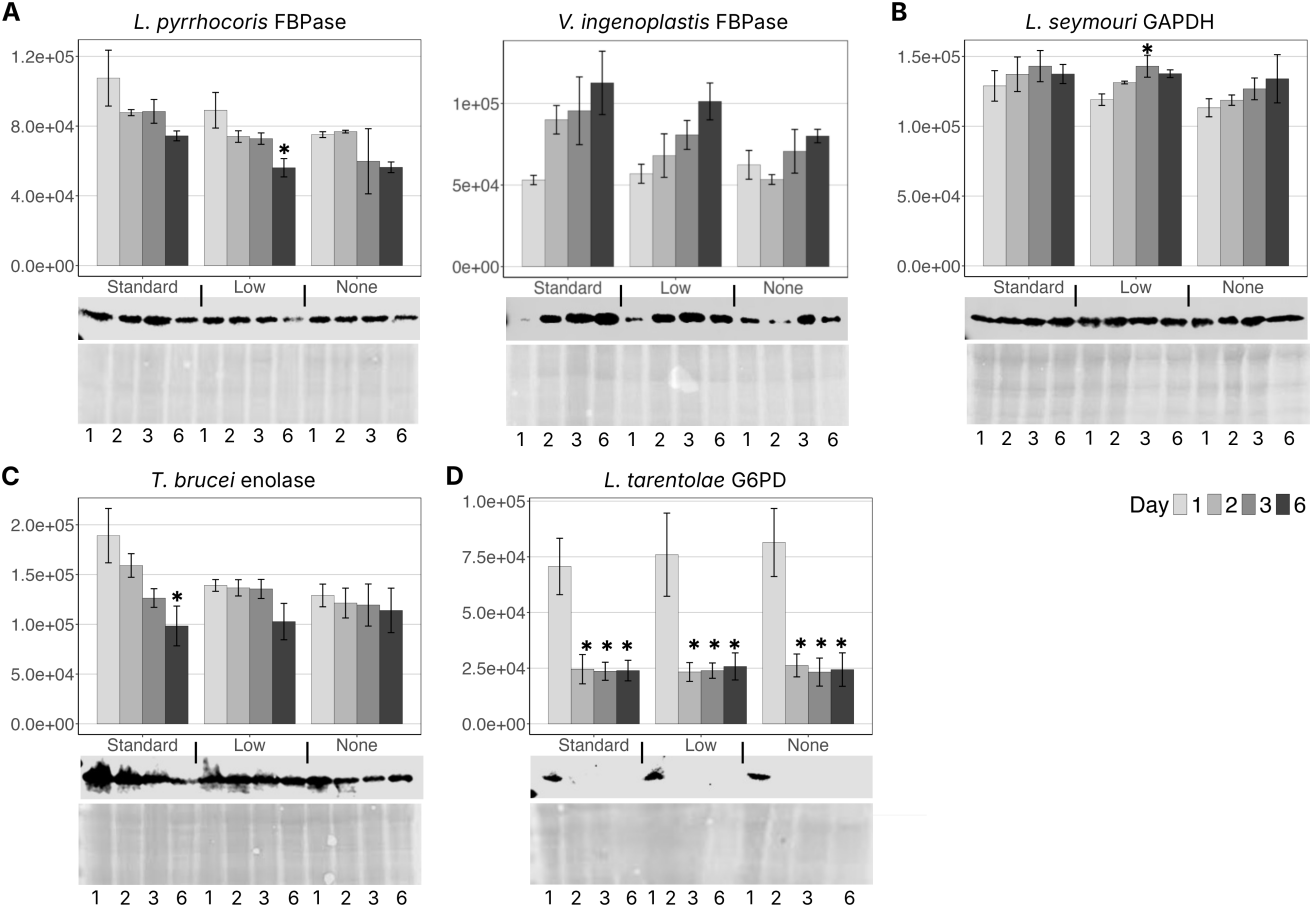
Enzyme abundances of *L. pyrrhocoris* (left) and *V. ingenoplastis* (right) FBPase **(A)**, *L. seymouri* GAPDH **(B)**, *T. brucei* enolase **(C)**, and *L. tarentolae* G6PD **(D)**, changing at time points over six-day growth. Enzyme abundance was determined in parasites grown in three LIT media formulations with added glucose of 2 g/L, 0.4 g/L and 0 g/L (Glucose-rich Standard, Low, and None, respectively). The top panels show densitometric analysis of immunoblot images of the indicated enzymes in the indicated species in two biological replicates for FBPase **(A)** and three biological replicates for the other plots. The x-axis denotes protein sample collection day and the y-axis the normalized integrated density of signal from each protein. Error bars represent the standard error of the mean (SEM). Enzyme abundance values at Day 2, 3, and 6 were each compared back to abundance on Day 1. Statistical significance was determined using one-way ANOVA followed by Dunnett’s *post hoc* test, with * indicating p-values less than 0.05. The middle panels are representative images of the immunoblots analyzed, while the bottom panels are corresponding images of Ponceau S staining of the blot used for within-gel sample loading normalization.

Among many unchanged glycosome enzyme abundances, there were some species and condition-dependent trends. FBPase abundance in *L. pyrrhocoris* trended down, while the presumed FBPase in *V. ingenoplastis* trended up during the 6-day time course (**Figure 3A**), and *L. seymouri* GAPDH abundance exhibited a gradual increase in days 2 and 3 (**Figure 3B**). In fact, the change was significant by Day 6 in the low glucose growth medium for *L. pyrrhocoris* FBPase and at Day 3 for *L. seymouri* GAPDH in the same medium (**Supplementary Table 1**). Trends in **Figures 3A, B** were mostly consistent regardless of the starting concentration of glucose in the medium, suggesting that overall nutrient depletion is responsible for these species-dependent alterations to glycosome enzyme abundances. However, the fact of these enzymes’ compartmentalization may be incidental, as there are also examples of cytosolic enolase apparently responding to medium nutrient changes over 6 days of culture growth in *T. brucei* (**Figure 3C**). This abundance change is significant at Day 6 and seems dependent on loss of glucose, as the trend is less apparent when cells are grown with low or no glucose. Finally, a singularly striking, glucose-independent significant decrease in *L. tarentolae* G6PD is apparent by Day 2 (**Figure 3D**) that could indicate changes in a cytosolic or glycosomal pool, or both. Changes in G6PD are not evident in any other species.

**Figure 4:**
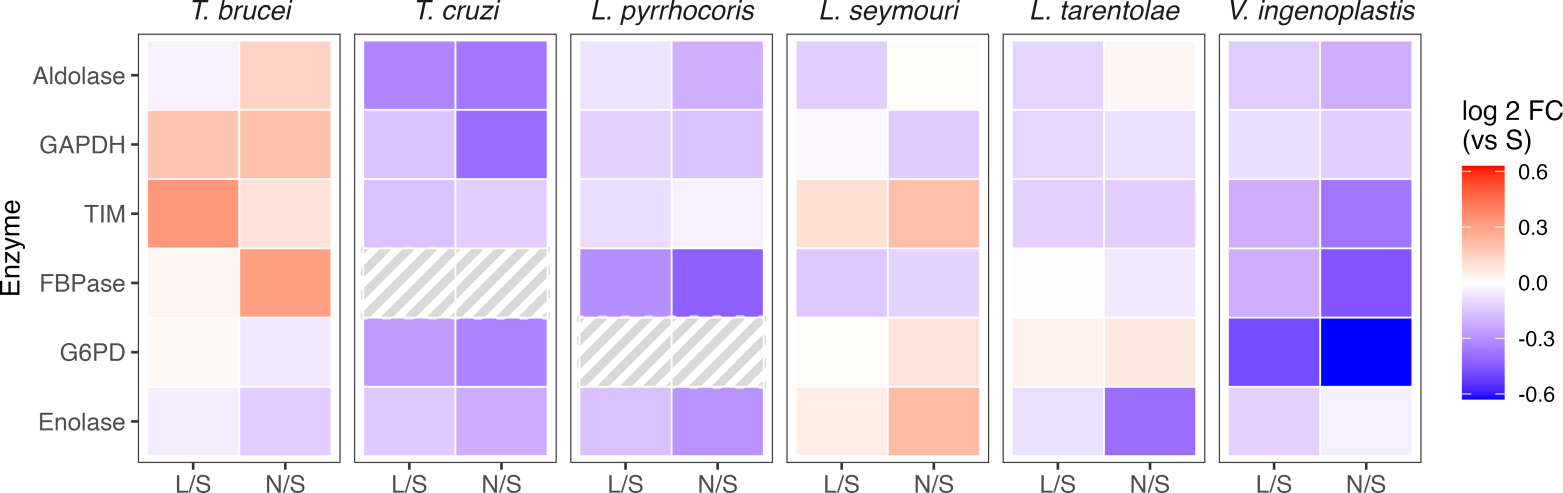
Enzyme abundance during six-day growth in media containing differing amounts of starting glucose. Area under the curve (AUC) was determined for normalized integrated density of bands detected by each specific antibody on the y axis relative to time on the x axis, in immunoblots analyzed in **Supplementary Figure 2**. Two comparisons of relative AUCs for growth in the different LIT media were made: Low (0.4 g/L) relative to Glucose-rich Standard (2 g/L) amounts (L/S), and None relative to Standard (N/S). Heat maps indicate relative amounts of the indicated enzyme in the positive (red) or negative (blue) direction for each comparison. Hash marks indicate experiments were not performed for the enzyme and indicted species.

We also wanted to know if parasites exhibited differences in enzyme abundances among standard, low, or no glucose medium growth. To do this we calculated area under the curve (AUC) for each plot of quantitative abundance during six-day culture growth (**Supplementary Figure 3**). Ratios of AUC between low and standard, and no and standard glucose were then plotted on a heat map to identify trends (**Figure 4**). Two species had the most consistent responses across enzymes and regardless of whether low or no glucose was being compared. These were *T. cruzi* and *V. ingenoplastis* where most enzymes were decreased when grown in glucose-deprived environments. In contrast, a number of enzymes were mildly increased in *T. brucei* in the same comparisons. *L. tarentolae* glycosome enzymes were virtually unaffected by presence or absence of glucose in growth media. Overall, AUC ratios never differed more than 0.6-fold increased or decreased, so these responses were extremely modest. We also compared enzyme abundances at each specific collection time point between the three different media using Tukey’s Honest Significant Difference (HSD) test, α = 0.05, with statistically significant differences in protein concentrations reported in **Table 1**. Significant differences appeared on specific days between growth in different media in *T. cruzi*, *L. pyrrhocoris*, *L. seymouri* and *L. tarentolae*. However, the magnitude of these differences was small and never occurred on more than one day of comparison. In summary, the magnitude and patterns of differences suggest that glucose availability has minimal impact on remodeling of these metabolic enzymes in the tested species.

**Table 1:** Statistically significant differences in enzyme abundances among parasites grown six days in LIT media formulations with added glucose of either 2 g/L (Standard), 0.4 g/L (Low) and 0 g/L (None), tested at Day 2, Day 3, and Day 6 collection time points.

Species	Enzyme	Day	n	Standard	Low	None
*T. cruzi*	Aldolase	3	3	A	A, B	B
*T. cruzi*	GAPDH	6	3	A	A, B	B
*L. pyrrhocoris*	TIM	1	3	A	B	B
*L. pyrrhocoris*	FBPase	2	2	A	B	A, B
*L. seymouri*	Enolase	3	3	B	A, B	A
*L. tarentolae*	Aldolase	1	2	A	B	A, B
*L. tarentolae*	GAPDH	1	2	A	A, B	B

Groups that share a letter are not statistically significantly different, as assessed by *post-hoc* Tukey’s Honest Significant Difference test following a one-way ANOVA, α= 0.05.

### Nutrition replenishment has some effect on glycosomal enzyme abundance

**Figure 5:**
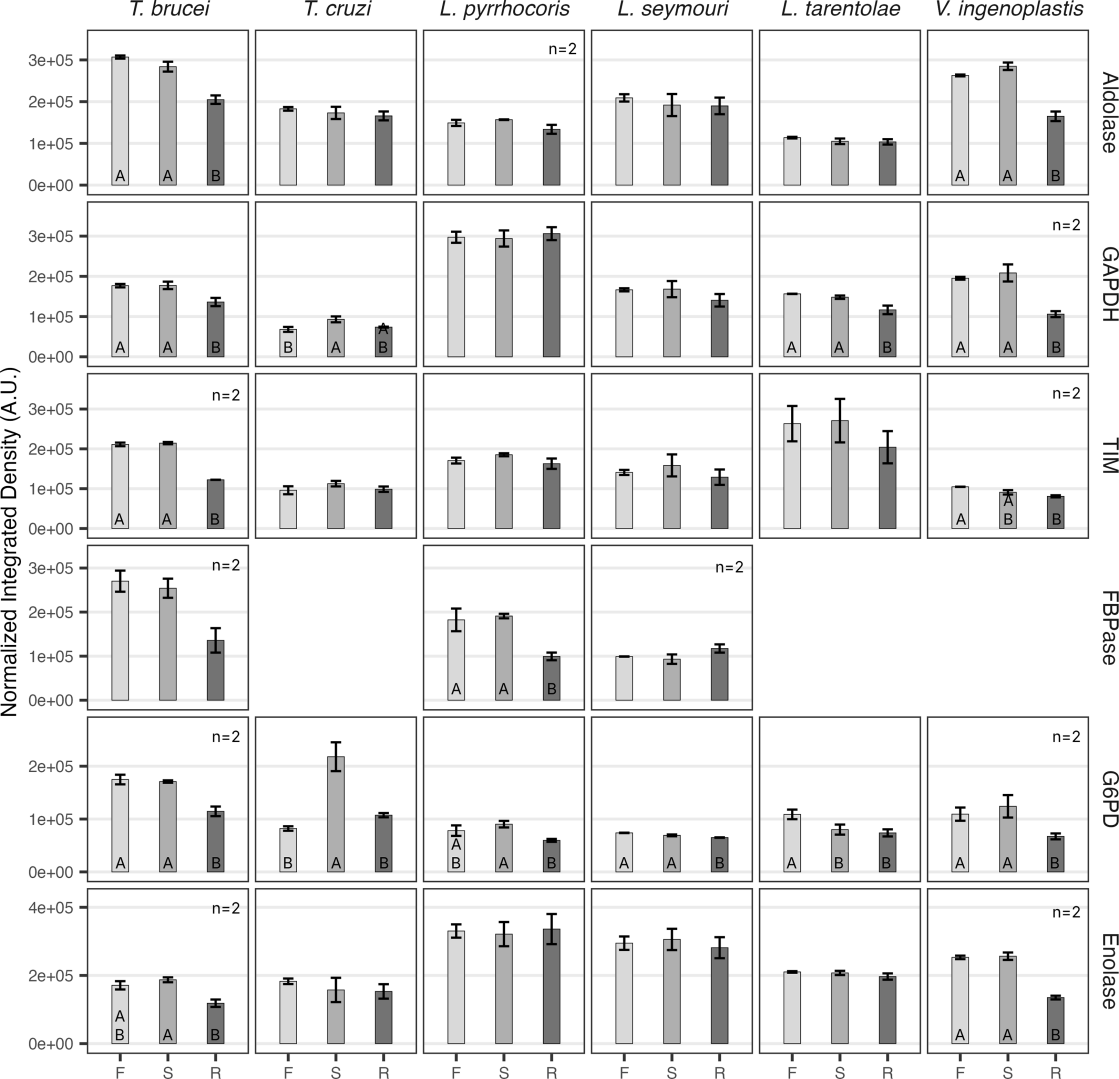
Enzyme abundance response to rapid restoration of nutrients. Enzyme abundance was determined in parasites grown in LIT medium lacking glucose from fed (F; 24 hours after transition to fresh medium), after 6-days’growth (starved, F), and 24 hours after replenishment with LIT medium containing the standard amount of glucose (R). The panels show densitometric analysis of immunoblot images of the indicated enzymes in the indicated species in three biological replicates unless indicated. The x-axis denotes protein sample collection day and the y-axis shows the normalized integrated density of signal from each protein. Error bars represent the standard error of the mean (SEM). Different letters on the bars denote differences in protein abundance as assessed by Tukey’s honest significant difference test following a one-way ANOVA, α = 0.05. Groups that share a letter are not statistically significantly different. FBPase abundances for *T. cruzi*, *L. tarentolae*, and *V. ingenoplastis* were not determined due to the challenging nature of utilizing the FBPase antibody in these species in these conditions.

Results of the previous experiment inferred that global glycosomal protein abundance is not predominantly modulated by the availability of glucose or gradual nutrient depletion in kinetoplastids. However, it is likely that all of these organisms undergo periodic fluctuations in nutrient levels, oscillating between abundance and scarcity (Hannaert et al., 2003). So, we also determined the impact of restoration of nutrients to starving parasites on the abundance of their glycosome-localized enzymes. All the organisms were acclimated to the no glucose medium for the start of this experiment in the same manner as for the previous experiments. Protein was collected 24 hours post-inoculation in fresh glucose-deficient media, after six days of cultivation, and 24 hours after the reintroduction of glucose-replete standard media. We were most interested in enzyme levels in parasites at the final collection point relative to those of the previous or initial collection point and used Tukey’s HSD test following one-way ANOVA, α = 0.05, to determine how enzyme abundances at each collection points differed. Glycolytic enzyme abundances were largely unaffected by nutrient replenishment in *T. cruzi*, *L. tarentolae*, and both *Leptomonas* species. In contrast, these enzymes, regardless of their localization, were markedly decreased in *T. brucei* and *V. ingenoplastis* after feeding (**Figure 5**). FBPase and G6PD were also decreased in the *T. brucei* sample under nutrient replenishment that includes glucose; the putative G6PD was decreased as well in *V. ingenoplastis*. This result paired with the relative invariability of enzyme abundances during the preceding slow starvation for *T. brucei* and *V. ingenoplastis* suggests that both these species are uniquely sensitive to sudden restoration of specifically glucose to the environment. Additionally, G6PD appeared to be depleted in nearly all species once nutrients were restored, when compared to the Day 1 and/or Day 6 time points. Thus, for kinetoplastids in general, a common response to a sudden influx of glucose may be a reduction in G6PD, at least initially. This is interesting as G6PD abundance was initially anticipated to be relatively stable given its abundance patterns across *T. brucei* life stages.

### All species experience less growth when grown under lower ambient oxygen

In addition to availability of nutrients, oxygen availability might also influence glycosome enzyme composition, as products of glycosome-compartmentalized pathways can be further catabolized in respiration-linked processes, and some glycosome-localized enzymes are involved in scavenging reactive oxygen species. Low oxygen is a significant factor influencing the pathogen-host response, including that of trypanosomatids, within infected and inflamed tissues in mammals (Jantsch and Schödel, 2015). Trypanosomatids in their insect stages appear to lack the capacity for sustained growth without oxygen. Specifically, *Leishmania* promastigotes exhibit limited anaerobic function, entering a reversible metabolic dormancy in anoxic conditions (Van Hellemond and Tielens, 1997), while *T. cruzi* requires respiration for energy generation (Stoppani et al., 1980). Similarly, procyclic *T. brucei* ceases cellular division and begins to perish after a short period in oxygen-deprived environments (van Weelden et al., 2003). However, although insects have a mechanism to distribute oxygen and other gases throughout their bodies, the oxygen levels in the alimentary canal do vary (Johnson and V Barbehenn R, 2000; Wong et al., 2011; Jang et al., 2021) The aerobic fermentation that occurs in trypanosomatids (Tielens and van Hellemond, 2009) could be an adaptive mechanism for variations in oxygen availability in the insect vector.

**Figure 6:**
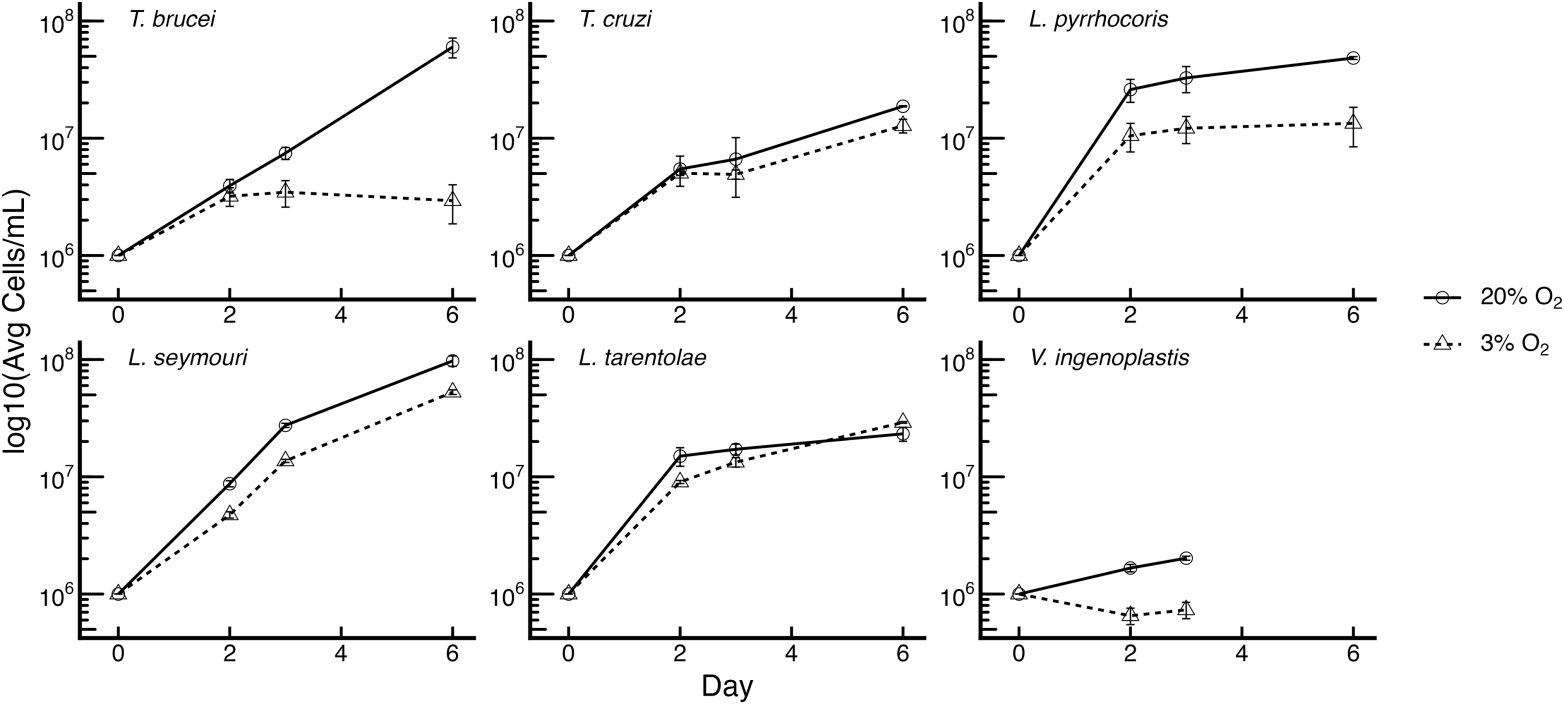
Parasite culture growth rates in low or ambient oxygen. The continuous growth of parasites starting from 1 x 10^6^ cells/ml was quantified in LIT medium lacking glucose. Flasks were placed in incubators with ambient air oxygen concentration (20%) or 3% oxygen. Error bars denote standard deviation. *V. ingenoplastis* can only grow for a limited time without glucose, and as the low numbers of cells at day 6 3% O_2_ also exhibited morphological aberrancies and may not have been alive, growth was only analyzed through three days of incubation.

As an initial determination of the sensitivity of species in culture to oxygen availability, we measured their proliferation in an incubator with ambient oxygen levels (~20%), or in a hypoxic chamber with oxygen level set at three percent. Parasite growth of cultures in both conditions was measured on the second, third, and sixth days. The experiment was performed a single time in standard LIT (not shown) and in LIT lacking glucose. Since results were more profound in medium lacking glucose, growth was measured in biological triplicate for this condition (**Figure 6**). Overall, growth trended lower in cultures placed in the 3% oxygen environment for all tested species, although the magnitude of the impact differed. *L. tarentolae* and *T. cruzi* showed the least difference in growth under different oxygen tensions. *V. ingenoplastis* cultures appeared dead by the 6-day time point, so growth comparisons for this organism were only made for three days of culture growth. As the data indicate that all examined species replicate more slowly in low oxygen conditions, the level of environmental oxygen may influence parasite metabolism or other cellular functions, including functions involving glycosome-compartmentalized enzymes.

### The expression of glycosome-localized proteins is not influenced by hypoxia

**Figure 7:**
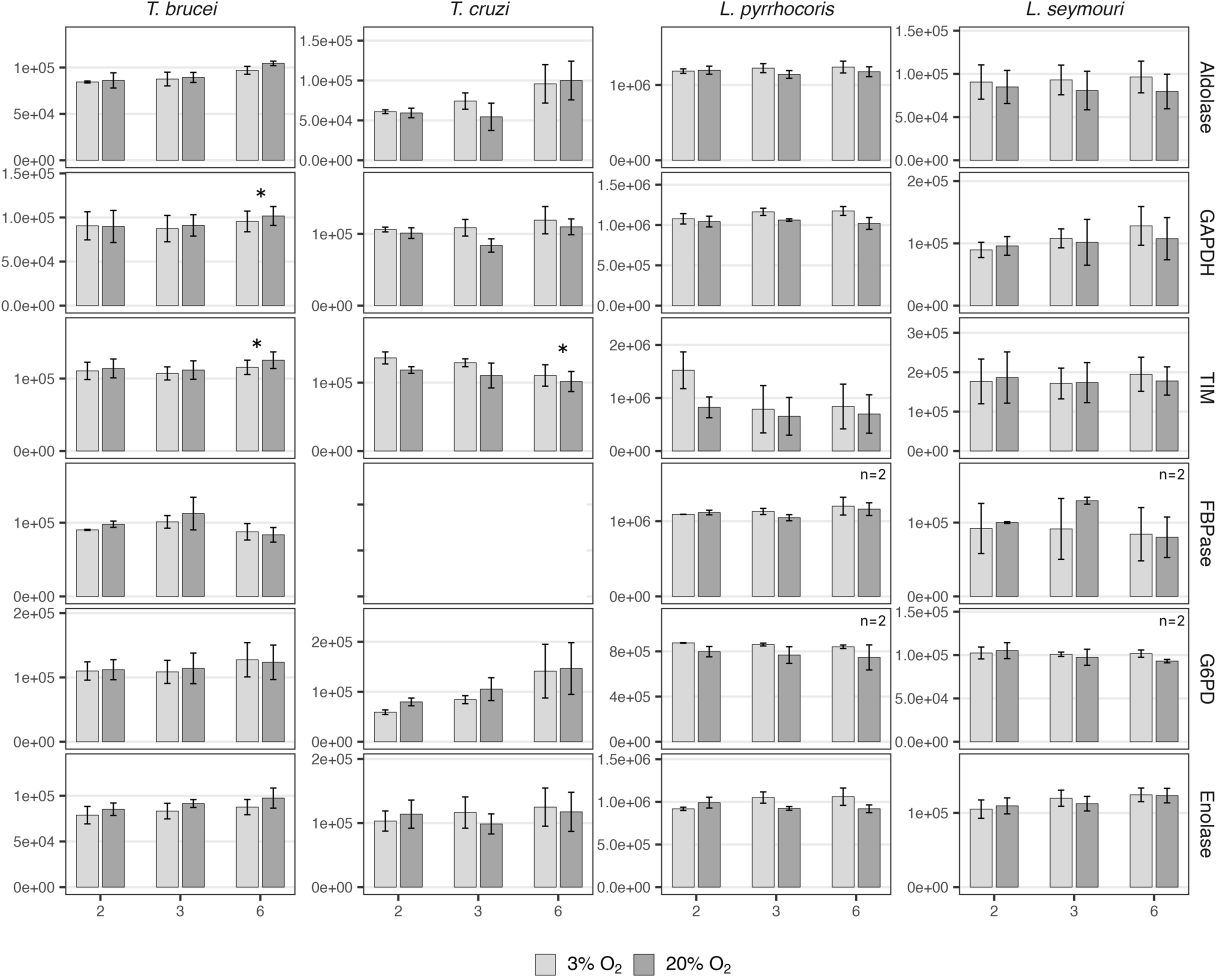
Enzyme abundance response to oxygen availability in selected trypanosomatid species. Enzyme abundance was determined in parasites grown in LIT medium lacking glucose over six days in incubators set to ambient (~20%) or 3% oxygen concentration. The panels show densitometric analysis of immunoblot images of the indicated enzymes in the indicated species in three biological replicates unless indicated. The y-axes show the normalized integrated density of signal from each protein at each day of growth in 3% or 20% air oxygen concentration, the x-axes indicating day of incubation. Error bars represent the standard error of the mean (SEM). Statistical significance of differences in enzyme abundance between oxygen concentrations at each day was determined using a two-tailed t-test, with * indicating p-values less than 0.05, 3% being compared to standard 20%. *T. cruzi* FBPase was not tested in replicate due to the challenging nature of its utilization in this experiment, and thus the panel was left off of the image.

We then determined whether oxygen tension serves as a glycosome composition remodeling catalyst using the same approach as used to explore the impact of nutrient availability. In initial experiments, we compared 3 and 20% oxygen environments on enzyme abundances for all six cultured kinetoplastid species grown in media with standard glucose levels or no glucose (not shown) except for *V. ingenoplastis*, for which the culture was not healthy enough for protein collection after 6 days growth in 3% oxygen. After one replicate, the potential for abundance differences appeared in immunoblots for only two species, and only when grown in medium lacking glucose. Specifically, glycosome-localized *Leptomonas* enzyme abundances were higher in the 3% oxygen environment. We subsequently performed replicate experiments only in *Leptomonas* and *Trypanosoma* species for representation of monoxenous and dixenous species. Significant differences between enzyme levels in the 3% and 20% oxygen environments were determined with a two-way t-test and a significance threshold set at a p-value of 0.05. While the initial experiment offered promising observations, aggregated data from three replicates yielded only minor trends or statistically significant changes in the abundance of glycosome-localized enzymes or cytosolic enolase (**Figure 7**). Nonetheless, the trend was that of metabolic enzymes slightly upregulated when oxygen is low in *Leptomonas* species, particularly at days 3 and 6, regardless of enzyme localization. The *Trypanosoma* species did not exhibit any patterns in its variations in enzyme abundance. The observation that *Leptomonas* enzymes are upregulated in a no-glucose, hypoxic environment compared to *Trypanosoma* species may reflect a metabolic adaptations specific to this genus, so that *Leptomonas* is better prepared to utilize any trace amounts of environmental glucose. In summary, it appears that abrupt fluctuations in nutrients influence the composition of the glycosome more than other tested perturbations. However, the abundances of tested enzymes were remarkably unresponsive overall.

## Discussion

The idea that the rapid remodeling of metabolic protein composition is a major function of the peroxisome-like organelle of Trypanosomatids and Diplonema was empirically addressed in this study. We reasoned that for this to be the case, we should be able to perturb abundances of the compartmentalized enzymes by altering extracellular signals in a single life stage, or for monoxenous species, similar to the way these enzymes are altered in abundance between the BSF and PCF of *T. brucei*. We intended to reveal patterns of protein abundance, if they existed, that would align with the hypothesis that glycosome enzyme compartmentalization is an efficient enzyme remodeling mechanism for kinetoplastids in general. Based on four proteins with likely glycosome matrix localization and one at least partially localized in six species (Durrani et al., 2020), the overall picture is instead of metabolic enzymes that are remarkably stable in overall abundance under the specific signals of differing nutrient or oxygen availability. For *T. cruzi*, results suggesting stability during epimastigote starvation in **Supplementary Figure 3** and **Figure 4** are consistent with a qualitative proteomic study suggesting low variation of the contents of purified glycosomes from exponentially growing and stationary phase (Acosta et al., 2019). Of course, parasite response to other external signals could yield major perturbations, particularly those that are also impactful in life stage transitions or varying between life stages, such as the presence of reactive oxygen species, differing temperature, or pH. Similarly, this study was specific to insect proliferative stages of both mono- and dixenous species. Whether the same tested metabolic enzymes of dixenous species’ second-host proliferative or transitional life stages are likewise as stable remains to be seen. Finally, only stability of pexophagy and glycosome biogenesis rates under conditions of changing nutrient and oxygen levels would truly prove a lack of impact of these perturbations on glycosome turnover.

One limitation of this study is that we are relying on localization studies in a few dixenous species plus *in silico* analysis (Durrani et al., 2020) to assign localization for the six proteins in our study for all species. Some glycosome matrix enzymes can be distinguished by the presence of a peroxisome targeting signal (Opperdoes and Szikora, 2006). One of these, PTS1, is a three amino acid tag on the C-terminus. However, the other, PTS2, is cryptic, and many *T. brucei* enzymes that have been definitively glycosome-localized have no obvious import mechanism at all (Güther et al., 2014). While cytosolic enolase does not have a PTS1 in any species, as expected, neither does *T. brucei* G6PD, while *T. brucei* TIM targeting relies on an internal 22 amino acid sequence (Galland et al., 2010), and trypanosomatid aldolases are targeted through a PTS2 (Andrade-Alviárez et al., 2022; Chudzik et al., 2000). Therefore, we cannot rely on PTS1 or PTS2 as a hallmark of glycosome matrix proteins in this study. We did determine that the PTS1 of *T. brucei* GAPDH and FBPase was conserved by evaluating its sequence in one monoxenous species (*L. seymouri*). We also found using our algorithm developed previously (Durrani et al., 2020), that G6PD, with a below-confidence C-terminal amino acid sequence in *T. brucei*, appears to have a PTS1 above the confidence limit in *L. seymouri*. Regardless, we cannot rule out that one or more enzyme that is glycosome-localized (or partially so) in *T. brucei* and *Leishmania* may be differently localized in one or more species. In the future it will be important to rigorously analyze the degree of conservation of glycosome matrix enzymes experimentally by determining the glycosome composition in a monoxenous species. However, the fact that the enzymes analyzed here exhibit fairly consistent expression regardless of environmental perturbation makes the question of their localization a bit less relevant.

We had originally postulated that if glycosome pexophagy and neogenesis were utilized for more efficient cellular protein remodeling during transitions, glycosome localized proteins would exhibit faster or more dramatic abundance differences than proteins of the same or similar pathways localized in the cytosol. Therefore, another constraint of our study was the reliance on a single cytosolic enzyme as a comparator. In fact, a major disadvantage of relying on immunoblotting to track protein abundance is the few antisera that proved functional across all or most analyzed species. The inclusion of additional cytosolic and glycosomal enzymes would have enriched our analysis, providing more opportunity to identify contrasts between glycosomal and cytosolic enzyme abundance changes, few as these changes appear to be. We note that trypanosomes and *V. ingenoplastis* responded to replenished nutrients by generally downregulating abundances of enolase just as much as the glycosome enzymes we tracked. Thus, this representative cytosolic enzyme does suggest that the mechanism of this response does not center around glycosome pexophagy and neogenesis. A quantitative proteomic analysis of parasite responses to these and other stimuli would be the best way to really define them, which would have to be done organism-by-organism and is beyond the scope of this survey study.

The most dramatic change in enzyme abundance we observed was a marked depletion of G6PD that shunts glucose-6-phosphate from glycolysis to the PPP in *L. tarentolae* starting 2 days after the transition to fresh medium that initiates our experiments. This occurred regardless of the starting level of glucose in the medium (**Figure 3D**), thus this response is driven by depletion of nutrients in general rather than glucose specifically. Once nutrients have depleted to a certain level by Day 2, no additional depletion occurs. Interestingly, this time frame corresponds to *L. tarentolae* exiting the exponential growth phase in all three media. The PPP generates NADHP and the nucleotide synthesis precursor ribose-5-phosphate, although additional products include some in common with glycolysis that can thus re-enter that pathway (Kovářová and Barrett, 2016). In culture, following exponential growth *L. tarentolae* may require fewer cellular building blocks and more flux through the glycolytic pathway. While this may also be true of the other tested species, *L. tarentolae* appears unique in possibly utilizing G6PD abundance to control this switch.

Conversely, G6PD abundance trended up under low oxygen for *Leptomonas* species. Upregulation of this or other PPP enzymes may be useful in low oxygen to maintain or increase the production of NADPH for reductive biosynthesis and combating the mitochondrial-induced oxidative stress (Ghosh et al., 2015) possible under these conditions. NADPH generation through the PPP in parasites is thought to provide reducing power to counteract the oxidative stress that can be part of a host’s antimicrobial defense, although its importance may be species and life stage dependent. For example, G6PD exhibits expression correlated with oxidative stress levels across *T. cruzi* life stages (Igoillo-Esteve and Cazzulo, 2006), while in *Leishmania donovani*, overexpression of G6PD renders the cells resistant to reactive oxygen species (Mukherjee et al., 2013). However, this observation is inconsistent with findings in *Leishmania major* promastigotes, where glycolytic flux increases as the partial oxygen pressure (pO_2_) drops from atmospheric levels (95% O_2_) to 6% O_2_, although this effect reversed once oxygen levels dropped further (Keegan and Blum, 1990). The glycosome localization of glycolytic and PPP enzymes, and the positioning of the PPP between a parasite’s metabolic needs and stress responses may certainly be related, and is worthy of additional study across kinetoplastids.

An important caveat is that the PPP, like other compartmentalized pathways, is not confined to the glycosome. Only a portion, 10-40%, of G6PD activity is glycosome-localized in procyclic *T. brucei* (Heise and Opperdoes, 1999), and while subcellular localization experiments have confirmed the presence of this and other PPP enzymes in glycosomes, many of their activities are also found in the cytosol, circumstance-dependent (Kovářová and Barrett, 2016). Glycosomal targeting sequences have been bioinformatically identified on many PPP enzymes in a range of kinetoplastids, suggesting that they are also compartmentalized at least to some degree (Durrani et al., 2020). Specifically for G6PD, the finding described above regarding a possible “weaker” PTS1 across species may reflect a capacity for dual localization. Regardless, our work does not distinguish whether the differences we observe in G6PD abundance reflect what is occurring in the cytosolic or glycosomal pool, or both. Considering the bidirectional changes to G6PD that we observed during states of stress (nutrient and oxygen limitation), it would be useful to explore the flexibility of its spatial organization as well as that of other PPP enzymes across kinetoplastids. Delving into the potential dual localization of G6PD and other elements of the PPP may illuminate potential evolutionary advantages conferred by optional glycosomal compartmentalization.

Finally, we considered what the unresponsiveness of the tested metabolic enzyme abundances to dramatically changing environmental nutrient and oxygen availability might mean, especially as these environmental stimuli were substantial enough to moderate growth. Our conclusion is that post-translational modifications such as phosphorylation, may play a major role in metabolic remodeling of the tested kinetoplastid species in response to these stimuli. Given that kinetoplastids are known to undergo extensive post-translational modifications (Rosenzweig et al., 2008a; Zhang et al., 2020), this may be a predominant component of their response to environmental availability of resources, an idea to test with these and additional stimuli. Approaches such as mass spectrometry may be warranted in order to understand the degree to which posttranslational modifications are responsible for modifications to parasite metabolic flux, and this is a compelling future question.

## Data Availability

The original contributions presented in the study are included in the article/**Supplementary Material**. Further inquiries can be directed to the corresponding author.
