# Supplementary material for: Cross-species comparisons in a unified medium suggest broadly stable glycosome-linked enzyme levels under nutrient and oxygen variation
Source: Front Parasitol. 2026 Jun 15;5:1823935. doi: 10.3389/fpara.2026.1823935 (PMC13311094; doi:10.3389/fpara.2026.1823935)
Supplement: Supplementary file 1 [file Image1.pdf]

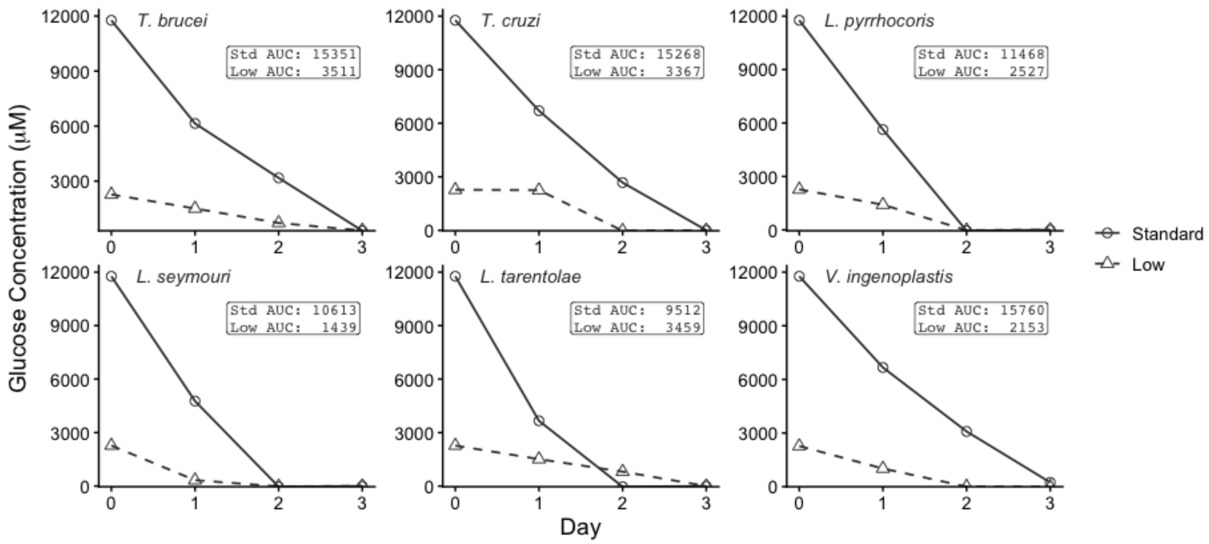

Supplementary Figure 1. Measured medium glucose concentration during the growth of each indicated parasite in LIT media formulated with Standard (Std; 2 g/L added glucose, solid line), or Low (0.4 g/L added glucose, dashed line) glucose. Cultures were started with 1 x 10^6 cells/ml (Day 0). The mean calculated in Figure 2 was used for the Day 0 time point. At other collection time points, data presented is a single replicate used to determine appropriate sampling time points for experiments. AUC, Area under the curve (μM x day).
