# Supplementary material for: Cross-species comparisons in a unified medium suggest broadly stable glycosome-linked enzyme levels under nutrient and oxygen variation
Source: Front Parasitol. 2026 Jun 15;5:1823935. doi: 10.3389/fpara.2026.1823935 (PMC13311094; doi:10.3389/fpara.2026.1823935)
Supplement: Supplementary file 2 [file Image2.pdf]

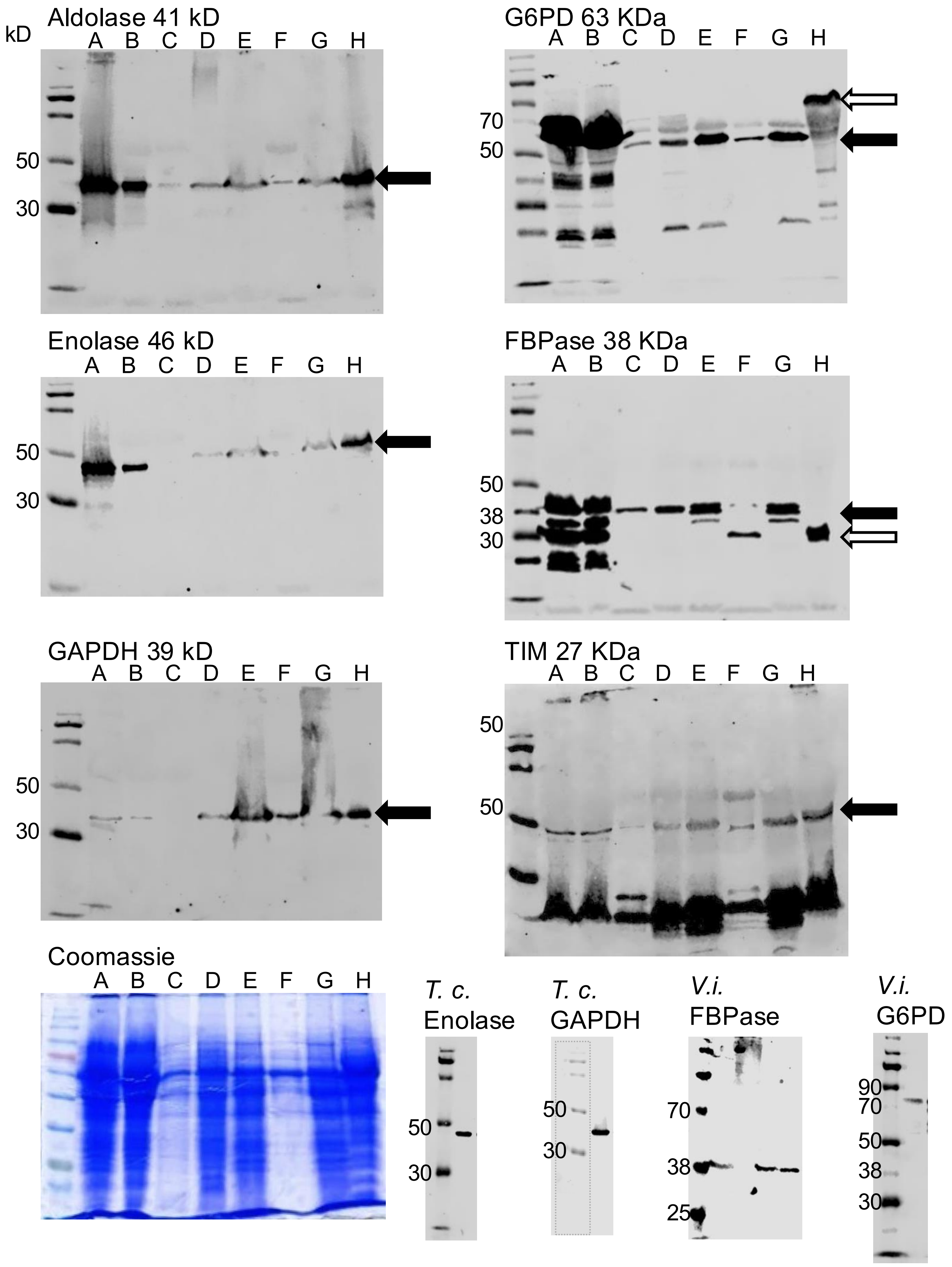

Supplementary Figure 2. Antibody cross reactivity among trypanosomatid species. Full immunoblot images of existing antibodies raised in rabbit against the *Trypanosoma brucei* homologue for each protein, except the GAPDH antibody raised against the *Leishmania mexicana* homologue. Loaded is total protein, amounts shown on the Coomassie image, for the following species/strains: A, *T. brucei* 29-13; B, *T. brucei* Eatro; C, *Trypanosoma cruzi*, D, *Leptomonas pyrrhocoris*; E, *Leptomonas seymouri*; F, *Leptomonas barvae* (not utilized further in the present study); G, *Leishmania tarentolae*; H, *Vickermania ingenoplastis*. Above each blot the predicted protein size to the nearest kD of the *T. brucei* homologue is indicated. In some cases, signal is overly intense in the *T. brucei* protein lanes, in order to visualize the bands from other species. Black arrows indicate band corresponding to the protein of interest. White arrows indicate major protein identified in *V. ingenoplastis* at a size inconsistent with the canonical protein size. Bottom middle: Additional immunoblots to demonstrate cross-reactivity of enolase and GAPDH antibody with *T. cruzi* protein that was insufficiently loaded in original experiments. Bottom right: Immunoblots demonstrating that while in additional assays, *V. ingenoplastis* FBPase size was found to be consistent with its homologues, G6PD is about 20 kDa larger.
