# Supplementary material for: Cross-species comparisons in a unified medium suggest broadly stable glycosome-linked enzyme levels under nutrient and oxygen variation
Source: Front Parasitol. 2026 Jun 15;5:1823935. doi: 10.3389/fpara.2026.1823935 (PMC13311094; doi:10.3389/fpara.2026.1823935)
Supplement: Supplementary file 3 [file Image3.pdf]

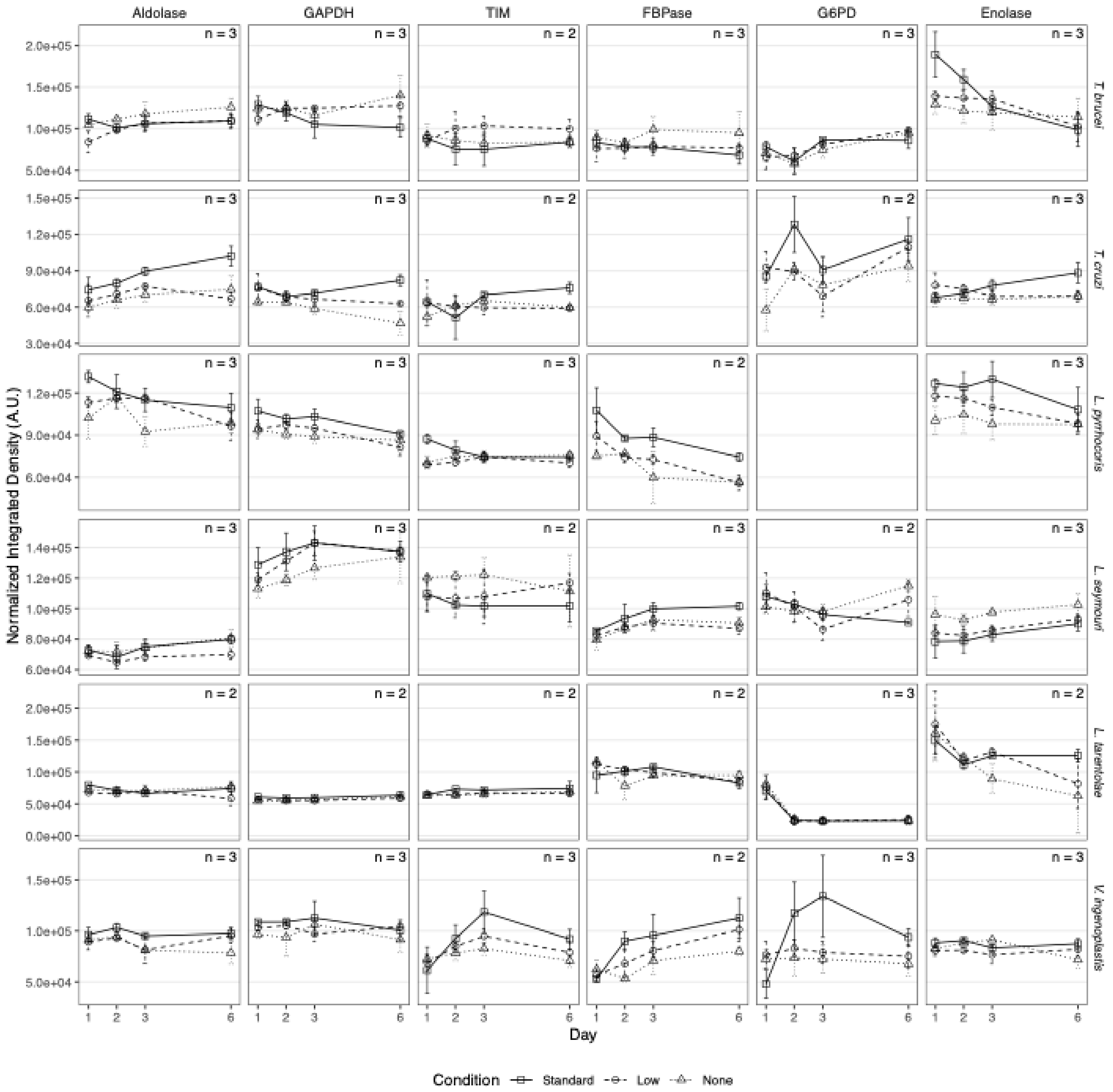

Figure S3. Enzyme abundance at each indicated day of a six-day growth period in LIT media formulated with Standard (2 g/L added glucose, solid line), Low (0.4 g/L added glucose, dashed line), or no added glucose (None, dotted line). The enzyme abundance for each measurement (y axis) is represented with the normalized integrated density of bands detected by each specific antibody on immunoblots. A.U., Absorbance Units. Error bars represent standard error. Plotted are mean values of two or three biological replicates as indicated. Antibody cross-reactivity was weak for *T. cruzi* FBPase and *L. pyrrhocoris* G6PD.
